# Automated Identification of Surgical Site Infections From Electronic Medical Records: Retrospective Observational Predictive Modeling Study

**DOI:** 10.2196/87896

**Published:** 2026-06-26

**Authors:** Arjun Chakraborty, Peter Tarczy-Hornoch, Dustin Long, Meliha Yetisgen

**Affiliations:** 1 Department of Biomedical Informatics and Medical Education, School of Medicine, University of Washington, 222 15th Street SW, Rochester, MN, 55902, United States, 1 5107095904; 2 Department of Pediatrics, School of Medicine, University of Washington, Seattle, WA, United States; 3 Department of Computer Science and Engineering, Paul G. Allen School of Computer Science and Engineering, College of Engineering, University of Washington, Seattle, WA, United States; 4 Department of Anesthesiology and Pain Medicine, School of Medicine, University of Washington, Seattle, WA, United States

**Keywords:** electronic health record, machine learning, natural language processing, surgical wound infection, epidemiology, diagnosis, neural networks computer, deep learning, large language models

## Abstract

**Background:**

Surgical site infections (SSIs) affect 160,000 to 300,000 patients annually, increasing postoperative mortality, causing significant complications, and incurring US $3.5 to US $10 billion in excess costs each year. Effective SSI surveillance can inform strategies to mitigate these outcomes. Traditional SSI surveillance methods, primarily manual chart reviews, are costly and labor-intensive.

**Objective:**

This study aimed to evaluate whether an automated SSI surveillance system built using newer natural language processing methods and deep learning could outperform previous approaches and whether such an approach could enable more efficient infection surveillance.

**Methods:**

Our dataset comprised approximately 30,000 surgical cases from the University of Washington Medical Center (UWMC) and Harborview Medical Center (HMC). Data from UWMC were captured for the National Surgical Quality Improvement Program, and data from HMC were captured for the National Healthcare Safety Network. Electronic health record (EHR) data for each surgical case included structured data pertaining to surgical procedure characteristics, laboratory values, and antibiotic administration, as well as clinical text notes for a surgical case from 7 days before to 90 days after surgery. Using this data, we applied a myriad of machine learning approaches to the task of SSI prediction. We reported the following performance metrics: *F*_1_-score, precision (positive predictive value), recall (sensitivity), area under the precision-recall curve, and precision at 0.9 recall for each machine learning approach.

**Results:**

In a cohort of 5996 surgical cases, incorporating multimodal EHR information—including contextual information from clinical text and temporal information from laboratory values—improved SSI prediction performance. Models using structured data and clinical text outperformed structured data alone (*F*_1_=0.68, 95% CI 0.68‐0.69 vs *F*_1_=0.55, 95% CI 0.54‐0.56; *P*<.001). Adding temporal features further improved performance (*F*_1_=0.70, 95% CI 0.69‐0.71; *P*<.001). Deep learning approaches leveraging large language models also outperformed the state-of-the-art rule-based system (*F*_1_=0.70, 95% CI 0.69‐0.71 vs *F*_1_=0.43; *P*<.001). The optimal approach combined foundation models for text summarization with deep learning methods for clinical text and temporal data processing. This system achieved a precision of 0.38 at 0.9 recall, demonstrating its potential for efficient, data-driven SSI surveillance.

**Conclusions:**

Automated surveillance approaches—particularly deep learning approaches—in combination with voluminous, multimodal data from the EHR, can enable more efficient infection surveillance processes. This has the potential to increase the quantity of SSI surveillance data available to guide interventions aimed at reducing SSI rates.

## Introduction

### Background

Surgical site infections (SSIs) are postsurgical complications that increase the risk of death and other secondary complications while substantially increasing overall health care costs [1,2]. SSIs extend the average length of hospital stay by 9.7 days and incur excess costs of approximately $20,000 per case [3]. The total cost of managing SSIs in the United States each year is estimated at $3.5 to $10 billion [4].

Surveillance of SSIs is essential for identifying infection trends, guiding prevention strategies, and benchmarking health care quality across institutions [5,6]. However, current surveillance practices rely heavily on manual chart abstraction, in which trained infection prevention personnel review electronic health records (EHRs) to determine whether a surgical case resulted in an SSI. This process is time-consuming, expensive, and difficult to scale, resulting in only a small proportion of surgical procedures being monitored [7]. Automated surveillance systems have been proposed to address this limitation by screening surgical cases using computational approaches prior to manual review, allowing infection prevention staff to focus on cases most likely to involve infection [8]. Most existing automated systems rely on rule-based algorithms or conventional machine learning (ML) methods applied to either structured clinical data (such as laboratory values) or unstructured clinical notes [9-13]. While these approaches can achieve high recall, their precision remains relatively low, meaning that multiple charts must still be reviewed to identify each true SSI case.

Recent advances in deep learning and natural language processing (NLP) offer opportunities to improve automated SSI surveillance. Clinical text and structured EHR data contain complementary signals relevant to infection detection, and temporal patterns in clinical data—such as changes in laboratory values over time—can provide additional diagnostic context. However, many prior approaches either exclude one of these modalities or rely on extensive manual feature engineering [9-13]. Deep learning models, such as convolutional neural networks (CNNs) and long short-term memory networks (LSTMs), can learn complex representations from multimodal and sequential data without extensive rule design. In addition, recent large language models (LLMs) and generalist foundation models have demonstrated strong performance across clinical NLP tasks, including summarizing clinical documentation. In this study, we developed an automated SSI surveillance system that integrates structured EHR data, clinical text, and temporal information using deep learning models and foundation model–based text summarization. We evaluated whether this multimodal, data-driven approach could improve the precision of automated SSI detection while maintaining high recall, thereby reducing the burden of manual chart review.

### Study Objectives

This study introduces and evaluates a multimodal, data-driven framework that integrates structured EHR data, temporal clinical signals, and foundation model–based clinical text representations using deep learning to improve the automated detection of SSIs. The focus of this study was on the retrospective identification rather than the prospective prediction of SSIs, with the purpose of improving the efficiency of surveillance methods. The main objectives of this study were as follows:

1. Apply one of the largest clinical datasets used to date for this task
2. Develop and assess a framework for how state-of-the-art NLP approaches, in combination with deep learning, can be used in a data-driven way to combine structured clinical text and temporal data to build highly performant post hoc SSI identification models (primary study emphasis)
3. Perform one of the first evaluations, to our knowledge, on the performance of approaches using foundational LLMs (eg, Llama 3) for this task
4. Produce a model that is more performant than previous methods on the task of post hoc SSI identification, potentially allowing for a substantial reduction in the workload of infection prevention staff.

## Methods

### Dataset

Our dataset included a subset of all surgical cases performed at the University of Washington Medical Center (UWMC) and Harborview Medical Center (HMC) between 2010 and 2020. The dataset comprised 29,980 surgical cases (n=1206, 4% SSI-positive cases and n=28,774, 96% SSI-negative cases) from 2 registries: data from UWMC were captured for the National Surgical Quality Improvement Program (NSQIP), and data from HMC were captured for National Healthcare Safety Network (NHSN). A total of 17,403 (58%) surgical cases came from UWMC and 12,577 (42%) from HMC.

To address differences in data capture frameworks (targeted vs broader surveillance), surveillance windows, and potential variation in documentation practices between NSQIP and NHSN, we standardized postoperative observation to a 90-day window and trained models on multimodal EHR data rather than clinical notes alone. Although integrating data across institutions and surveillance programs may reduce observed performance, this approach was intended to improve generalizability by incorporating broader clinical variability.

Our dataset represents one of the largest studies of its kind to date, featuring a diverse range of 7 procedure classes, including craniotomy, spine, orthopedic, general, gynecological, cardiothoracic, and vascular surgeries. Data from UWMC consisted of NSQIP “target” procedures in cardiothoracic, vascular, general, and gynecological surgeries (cardiac surgery, vascular surgery, pancreatectomy, hepatectomy, esophagectomy, colectomy, proctectomy, appendectomy, ventral hernia repair, bariatric surgery, gynecologic reconstructive surgery, hysterectomy, cystectomy, and thyroidectomy). Data from HMC consisted of colorectal surgery, abdominal hysterectomy, spinal fusion, craniotomy, ventriculoperitoneal shunt placement, and instrumented hip and lower-extremity procedures. Cases with infections present at the time of surgery were excluded. All classes of SSI (superficial, deep, or organ/space) within the procedure-specific 30- or 90-day surveillance period were included. We included transplant cases and other instances where patients may develop infections, despite having normal laboratory values and vital signs (eg, normal white blood cell [WBC] counts) due to immunosuppression. Each surgical case was assigned a binary value, indicating the occurrence of SSI according to NHSN or NSQIP criteria. We used EHR data to represent each case, incorporating static structured and temporal variables, as well as unstructured clinical notes.

### Data Representation

#### Structured Data Representation

Structured data elements included (1) surgical procedure characteristics, (2) laboratory values (eg, WBC counts and microbiology orders), (3) postoperative fever (temperature), (4) antibiotic administration events, and (5) postoperative consultations with infectious disease teams or interventional radiology for drain placement. The surgical procedure type was one-hot encoded as “Surgical Procedure Class,” and each case was labeled based on whether a reoperation occurred within 90 days (“Reoperation”).

Consultation variables were represented as binary indicators for postoperative infectious disease consultation (“ID Consult”) and interventional radiology drain placement (“IR Drain”) within 90 days. Additional binary indicators captured whether a culture order (“Culture Ordered”) or SSI-relevant antibiotic administration (“Antibiotic Administration”) occurred during the same period.

Continuous laboratory values and vitals (WBC counts and temperature) were summarized using their maximum values within the first 90 postoperative days. Outliers (eg, >100 k/mm³ for WBC or >50 °C for temperature) and missing values were replaced with the mean from the training set. All features were then min-max normalized prior to model input. For additional details on our structured data representation, see Multimedia Appendix 1.

#### Unstructured Clinical Note Representation

The clinical text corpus included all notes associated with each surgical case from 7 days before surgery to 90 days afterward (3,193,094 notes total). The most common note types included inpatient nursing notes, telephone encounters, progress notes, operative reports, and discharge summaries. Patient portal messages were not included in the unstructured notes. For each surgical case, all notes were concatenated into a single pseudodocument representing the case’s text data.

Preprocessing included lowercasing and, for discrete vectorization approaches, the removal of stopwords, numbers, and punctuation [14]. A key challenge was that SSI-related information is sparse and distributed across diverse note types and time points. To address this, we developed representations that selectively extract the information most relevant to SSI (“information extracted”).

We evaluated 4 text representation approaches:

1. Unigrams: Each word was treated as a feature using a bag-of-words representation with term frequency-inverse document frequency (TF-IDF) weighting [15]. Only words appearing in at least 5 documents were included. The top N features were selected using an ANOVA *F* test prior to model training, with N determined through a hyperparameter search.
2. Unified Medical Language System (UMLS) disease and chemical concepts: Disease and chemical concepts were defined using UMLS semantic groups [16] and identified with scispaCy (Allen Institute for AI) [17]. We retained the 200 concepts with the highest ANOVA *F* values relative to the SSI label. These concepts were represented using a bag-of-words TF-IDF approach, where document frequency was defined by the number of surgical cases containing the concept. The remaining TF-IDF implementation details matched the unigram approach.
3. Sentences containing UMLS disease and chemical concepts (information extracted): To capture contextual information, we extracted sentences containing identified disease or chemical UMLS concepts. Sentences were ordered temporally by note date and by their position within each note. To reduce noise, only sentences containing concepts appearing in fewer than 15% of pseudodocuments were retained. The resulting sentence sequences were vectorized using word embeddings trained on our dataset with the word2vec continuous bag-of-words architecture [18]. In a separate approach, we also used embeddings derived from ClinicalBERT, which incorporates domain-specific knowledge through pretraining and fine-tuning on clinical corpora [19].
4. Clinical note summaries (information extracted): We used Llama-3-70B-Instruct (Meta AI), accessed through the Hugging Face Transformers framework, to summarize clinical notes. The average length of each clinical note in our dataset was 1772 words. We condensed each note into a summary of 100 words. The manuscript authors, 2 of whom are clinicians, jointly reviewed the generated summaries to ensure they were consistent, reproducible, accurate, and without hallucinations. Using parallel graphics processing unit inference (4 parallel graphics processing units), the average processing time was approximately 1 second per note, and the full summarization process for our 3,193,094 notes was completed in about 9 days. These summaries for each clinical note were concatenated temporally in order of note date. Our summarization prompt was as follows: “Summarize the following clinical note emphasizing development of infection and administration of antibiotics for surgical site infection. Limit the summary to less than 100 words.” Our constrained prompt (“emphasizing development of infection and administration of antibiotics”) was designed to limit hallucinations. To capture information more salient to SSI prediction, we only summarized notes containing disease or chemical UMLS concepts. We vectorized the sequence of clinical note summaries using word embeddings. For additional details on our clinical text representation, see Multimedia Appendix 1.

#### Temporal Data Representation

To incorporate temporal dynamics, we extended the structured data by including time-stamped laboratory values (WBC and absolute neutrophil count) and vitals (temperature) from 7 days preoperatively to 90 days postoperatively. For temporal models, these variables were excluded from the static structured features.

Daily maximum values were computed and organized into fixed-length sequences, with shorter sequences padded with zeros. The sequence length (10 time points) was determined through hyperparameter tuning to balance model complexity with available data while capturing the clinically relevant perioperative window. Daily maxima were selected to emphasize peak physiological abnormalities associated with infection rather than averaging values, which may attenuate clinically meaningful signals.

**Figure 1.**
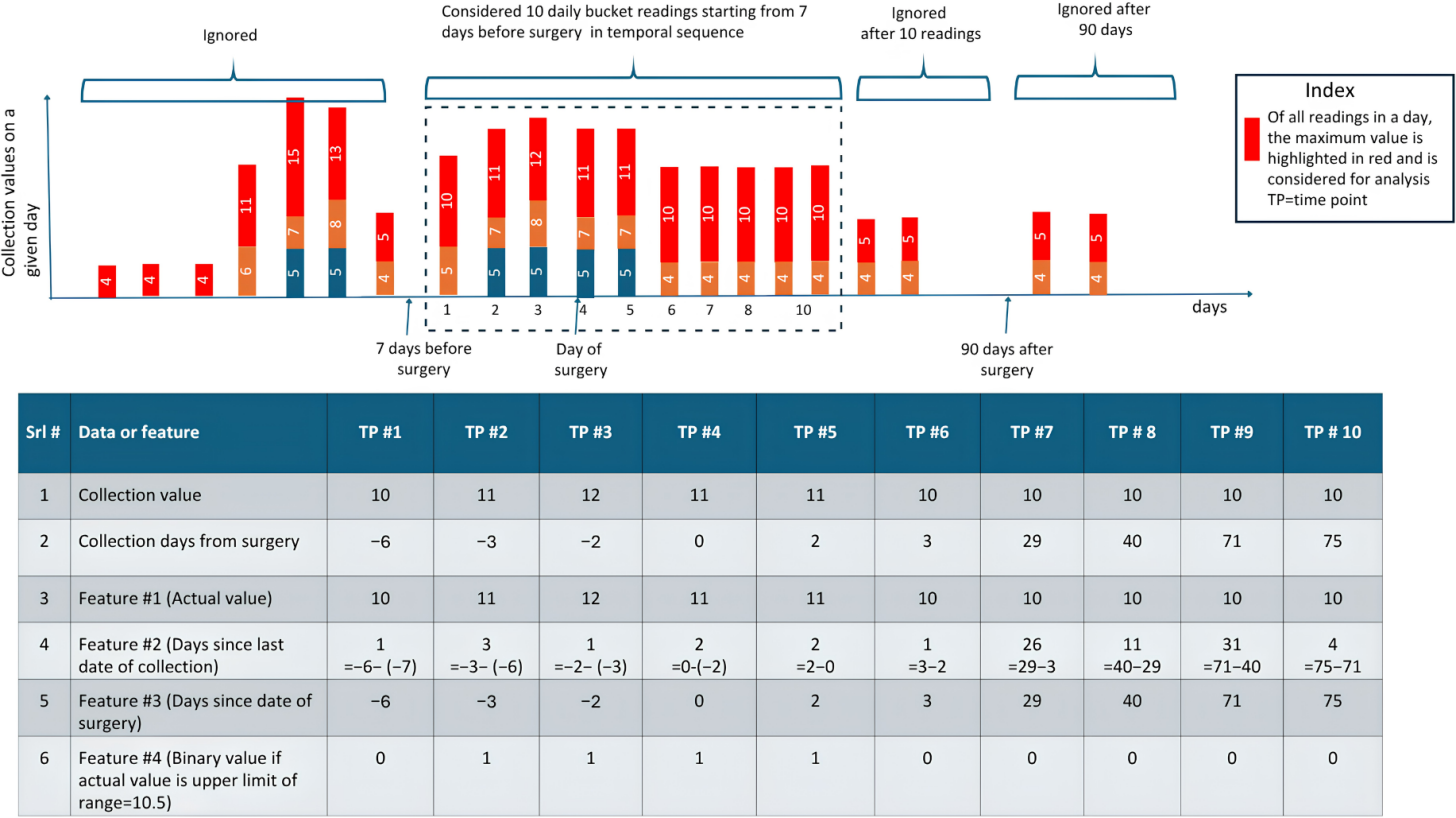
Method for constructing our temporal representation. Srl: serial TP: time point.

Zero padding was used to standardize sequence lengths and was informed by observed differences between SSI-positive and SSI-negative cases (Figure 1). In this dataset, SSI-negative cases generally exhibited fewer recorded measurements and lower overall values. As such, zero padding serves as a proxy for the absence of clinically significant events, rather than introducing artificial trends. The models were trained to interpret these padded values accordingly.

For each variable, 4 derived features were constructed: (1) fold change relative to the upper limit of normal, (2) time between consecutive measurements, (3) time since surgery, and (4) an indicator of whether the value exceeded the normal range. These features were generated from temporally ordered sequences and combined to form inputs to CNN and LSTM models. For additional details on our temporal representation, see Multimedia Appendix 1.

### SSI Classification Approach

We defined our SSI identification problem as a binary classification task. We defined 2 baselines and proposed several neural or deep learning–based approaches to solve the problem.

Baseline 1 (Rule-based algorithm): As the first baseline system, we used a rule-based system [9] that forms the basis of a published gold standard contemporary automated surveillance system [9]. This previously described rule-based system is available online [9]. It uses a text-processing pipeline with components including a section detector, named entity recognizer, and context detector to analyze clinical notes associated with surgical cases. We input each pseudodocument into this pipeline. The system produced a binary classification decision for each clinical text sample (pseudodocument), indicating whether SSI occurred or not after surgery.

Baseline 2 (Random forest): As the second baseline system, we developed a random forest (RF) model, which used either structured features alone or a combination of structured and unigram text features. RF was a strong baseline for this task, as many published works in the past have used this modeling approach [10,13].

Deep learning models: We compared the performance of the 2 baseline models against our neural or deep learning approaches. We constructed our static structured representation by inputting static structured features into a dense layer. In our feed-forward neural network models, we used this static structured representation alone or combined it with unigram features or UMLS concept features. Our deep learning models took UMLS concept sentences or Llama 3–generated clinical text summaries as input. These sentences or summaries were vectorized using either locally trained word embeddings or those generated by ClinicalBERT. The latent representations derived by different deep learning layers (CNN and bidirectional long short-term memory network [BiLSTM]) were concatenated with static structured data representations within our deep learning framework. In models incorporating temporal information, we input our temporal data representation into different deep learning layers (CNN and LSTM) to generate latent temporal representations. We concatenated these latent temporal representations with our best-performing (highest *F*_1_-score) text representation and our static structured data representation for training. Our neural and deep learning models included a dense layer and a final output layer following the concatenation layer.

The dataset was split into training, validation, and test sets in a 7:1:2 ratio. We used stratified training or validation or test splits to address the class imbalance in our dataset, in which only 4% (1206/29,980) of cases were SSI-positive. Because SSI is a relatively infrequent event (<5%), a class-weight parameter was applied during model training to account for the imbalance between SSI-positive and SSI-negative cases. We also explored alternative strategies to address class imbalance, including up-sampling SSI-positive cases through duplication and down-sampling SSI-negative cases through random removal. Interim analyses indicated that these approaches did not meaningfully affect model performance, and they were therefore not used in the final model development.

We systematically tested a range of hyperparameters for different model types using a grid-search technique as part of the tuning process to derive the optimal values. Hyperparameter tuning aimed to optimize the *F*_1_-score or the precision at 0.9 recall metric. We evaluated performance in each experiment using *F*_1_-score, precision (positive predictive value), recall (sensitivity), and area under the precision-recall curve. To measure performance, we also used a clinical utility metric (precision at 0.9 recall), which more closely reflects usefulness in a clinical use setting. One-sample 95% CIs based on the *t* distribution for each metric were reported.

We compared the performance achieved by an individual representation or strategy with another individual representation or strategy using a 2-tailed *t* test for the means of 2 independent samples. To control the family-wise error rate across multiple comparisons, we applied the Bonferroni correction, multiplying each *P* value by the total number of comparisons performed (10 comparisons for SSI prediction performance and 2 comparisons for clinical significance).

### Ethical Considerations

This study was reviewed and approved by the University of Washington Institutional Review Board (approval number: STUDY00005987). The requirement for informed consent was waived because the research involved a retrospective analysis of existing EHR data and posed minimal risk to participants. Access to the data was granted under the institutional review board approval, and all analyses were conducted in accordance with the University of Washington policies on human participants research and data protection.

Only authorized study personnel had access to identifiable information during data extraction. No direct patient contact occurred, and no participant compensation was involved. Privacy and confidentiality were protected through secure data storage on institutional, access-controlled servers, with all procedures following the Health Insurance Portability and Accountability Act (HIPAA) [20] and relevant institutional data-handling policies. All researchers and annotators completed human participants training and were authorized to handle data containing protected health information.

## Results

### Composition of Procedure Types and SSI Event Rates

The total number of cases and total SSI-positive cases for each procedure type are provided in Table 1. The SSI positivity rate in our dataset was 1206 out of 29,980 (4%).

**Table 1.** Surgery counts and infection rate.

Procedure class	Case count	SSI^a^ events rate, n (%)
Spine	3724	196 (5.3)
Orthopedic (nonspine)	2739	105 (3.8)
Neurosurgery (nonspine)	4990	185 (3.7)
General surgery	14,200	585 (4.1)
Gynecologic surgery	3211	120 (3.7)
Cardiothoracic surgery	328	8 (2.2)
Vascular surgery	788	7 (0.9)
Total	29,980	1206 (4.0)

a SSI: surgical site infection.

### Contributions of Model Type, Architecture, and Text Representation to Model Performance

We assessed the impact of using different feature sets, text representations, and modeling strategies on the task of SSI prediction. Table 2 summarizes the precision, recall, overall performance (*F*_1_-score), and area under the precision-recall curve of our different baseline comparators, feature sets, data representation methods, and modeling strategies. Our best-performing deep learning models (Table 2: Exp 11) outperformed RF models (Table 2: Exp 1 and 3). Integration of clinical text and structured data enhances the performance for both RF (Table 2: Exp 1 vs Exp 3) and neural models (Table 2: Exp 2 vs Exp 4 and 5). This supports our hypothesis that the incorporation of clinical text data adds new signals relevant to SSI prediction and thereby can enhance the performance of SSI prediction models. Using Llama 3 summaries outperformed using UMLS concept sentences (Table 2: Exp 7 vs Exp 9).

**Table 2.** Surgical site infection (SSI) prediction performance.

Experiment number	Model^a^	Features	Precision, mean (95% CI)	Recall, mean (95% CI)	*F*_1_, mean (95% CI)	AUPRC^b^, mean (95% CI)	*P* value	Comparison with experiment number
0	Rule-based model (baseline 1)	—^c^	0.34^d^	0.59^d^	0.43^d^	—	—	—
Structured only								
1	RF^e^ (baseline 2)	Structured only	0.64 (0.63-0.65)	0.42 (0.42-0.43)	0.51 (0.50-0.51)	0.53 (0.53-0.53)	—	—
2	FFNN^f^	Structured only	0.76 (0.76-0.76)^g^	0.42 (0.41-0.44)	0.54 (0.52-0.56)	0.57 (0.57-0.57)	.22	1
Multimodal								
3	RF	Structured+unigrams	0.69 (0.69-0.69)	0.47 (0.47-0.48)	0.57 (0.56-0.57)^h^	0.64 (0.64-0.64)	.005	1
4	FFNN	Structured+unigrams	0.58 (0.58-0.58)	0.67 (0.67-0.67)	0.62 (0.62-0.62)^h^	0.64 (0.64-0.65)	<.001	3
5	FFNN	Structured+UMLS^i^ concepts	0.60 (0.59-0.60)	0.67 (0.66-0.68)	0.63 (0.63-0.63)^h^	0.65 (0.64-0.65)	<.001	4
6	CNN^j^	Structured+UMLS sentences	0.62 (0.60-0.64)	0.64 (0.63-0.64)	0.63 (0.62-0.63)	0.63 (0.63-0.64)	—	5
7	BiLSTM^k^	Structured+UMLS sentences	0.62 (0.59-0.65)	0.67 (0.64-0.69)	0.64 (0.64-0.64)^h^	0.67 (0.67-0.67)	<.001	5
8	ClinicalBERT	Structured+UMLS sentences	0.61 (0.61-0.61)	0.66 (0.66-0.66)	0.63 (0.63-0.63)	0.63 (0.63-0.63)	—	5
9	BiLSTM	Structured+Llama 3 summaries	0.68 (0.68-0.69)	0.69 (0.69-0.70)	0.68 (0.68-0.69)^h^	0.74 (0.74-0.75)	<.001	5
Temporal methods								
10	BiLSTM or CNN	Structured+Llama 3summaries+temporal	0.66 (0.65-0.66)	0.66 (0.66-0.66)	0.66 (0.66-0.66)^h^	0.72 (0.72-0.72)	<.001	9
11	BiLSTM or LSTM^l^	Structured+Llama 3 summaries+temporal	0.69 (0.68-0.69)	0.71 (0.69-0.73)^g^	0.70 (0.69-0.71)^h^^,^^g^	0.78 (0.78-0.79)^g^	<.001	9

a Column values are formatted as text representation or temporal representation.

b AUPRC: area under the precision-recall curve.

c Not applicable.

d A 95% confidence interval is not applicable in this case, as the method is a nonstochastic rule-based system and therefore yields identical performance across runs, with no sampling variability to estimate uncertainty.

e RF: random forest.

f FFNN: feed-forward neural network.

g Highest precision, recall, *F*_1_, and AUPRC values.

h *F*_1 _values with statistical significance (*P*<.05).

i UMLS: Unified Medical Language System.

j CNN: convolutional neural network.

k BiLSTM: bidirectional long short-term memory network.

l LSTM: short-term memory network.

Using an LSTM layer to process temporal data outperforms using a CNN layer. The incorporation of temporal data with an LSTM layer thus improves overall performance, primarily driven by improvements in recall (Table 2: Exp 9 vs Exp 11). A plausible explanation for this is that the incorporation of temporal information on laboratory values and vitals added signals pertaining to SSI (eg, elevated laboratory values). Thus, it led to the correct classification of some previously false-positive cases that exhibit such signals.

### Factors Driving the Performance of Clinical Text Summaries Versus UMLS Concept Sentences

Our error analyses revealed that the following factors resulted in Llama 3–generated summaries (Table 2: Exp 9) outperforming UMLS concept sentences (Table 2: Exp 7):

1. Focusing our text representation on signals relevant to SSI: Our targeted summarization prompt (“emphasizing development of infection and administration of antibiotics for surgical site infection”) focused the clinical text summaries on information relevant to SSI prediction (eg, “treated with antibiotics for Klebsiella, *E. coli* …,” “the patient developed an abdominal wound infection after hernia repair surgery,” and “elevated white blood cell count”). This led to the model being able to focus on more salient information for SSI prediction (eg, signs and symptoms of, treatment for, and diagnosis of SSI) rather than being confused by less relevant information present in UMLS concept sentences (eg, “IVPB discontinued due to pain”).
2. Clinically irrelevant concept selection based on SSI correlation (part of the UMLS concept sentences approach): The UMLS concepts approach restricted sentence extraction to the top 200 concepts with the highest ANOVA *F* value between the concept and the SSI positivity label. This strategy helped avoid including sentences related to commonly recorded but irrelevant concepts, such as repeated mentions of the patient receiving a saline bolus. However, focusing solely on highly correlated concepts resulted in sentences enriched in positive cases but lacking clinical relevance to SSI (eg, “Renal insufficiency. Cr range of” and “chew Vitamin supplement”), which reduced the precision of Approach 2 for post hoc SSI prediction. In contrast, Llama 3, with its broad human biology knowledge and advanced reasoning capabilities, produced clinical note summaries rich in information essential for accurate post hoc SSI prediction.
3. Building more coherent text representations: Because our clinical text summaries were temporally ordered (by note date) and each contained information about one note, the resulting text representation was more coherent than one constructed using sentences containing UMLS concepts:

Summary: A [age] year old female patient...The patient had an abdominal exam worrisome for surgical site infection, with tenderness to palpation and erythema around an incision site ct abdomen/pelvis showed large amounts of abdominal subcutaneous gas, concerning for possible necrotizing soft tissue infection. ...summary: A [age] year old female patient was admitted to the hospital with symptoms of confusion, lethargy, and abdominal pain...a ct scan revealed free intra-abdominal air, extensive subcutaneous emphysema, and a possible abscess or leak.

This led to our BiLSTM models being able to process the information more effectively. In contrast, in the case of UMLS concept sentences, our BiLSTM models may have been confounded by abrupt chains of disjointed sentences with little connection to each other and lacking an overall logical message:

Allergies tylenol NSAIDs aztreonam Medications Nystatin topical as needed. Nystatin topical application Topical BID A CT was performed showing concern for abdominal wall abscess which was I&Ded initially with associated cellulitis. She was taken back to the OR today due to jejunostomy anastamosis leak and abdominal cellulitis that was repaired and debrided with no evidence of fasciitis.

[Problems Interventions Education Sepsis]

We have provided examples of false negatives and false positives to further illustrate these points in Multimedia Appendix 2 Multimedia Appendix 33.

### Clinical Significance

Assessment of the overall performance of our various approaches is not meaningful in actual practice if it does not reflect performance in a real-world clinical setting. In such a setting, our automated surveillance approach is likely to be used as a screening tool prior to manual chart review to rule out clearly negative cases. Thus, to assess the actual clinical importance of our various SSI prediction approaches, we assessed the precision of our models at a high (90%) recall (Table 3).

**Table 3.** Clinical significance of the various data representation and modeling approaches.

Experiment number	Model^a^	Features	Precision at 0.9 recall, mean (95% CI)	Number of charts reviewed per SSI^b^	Comparison with	*P* value
M	Manual review	—^c^	0.03^d^	33^d^	—	—
3	RF^e^	Structured+unigrams	0.17 (0.15-0.19)	5.9	—	—
5	FFNN^f^	Structured+UMLS^g^ sentences	0.26 (0.25-0.27)	3.8	—	—
9	BiLSTM^h^	Structured+Llama 3 summaries	0.35 (0.35-0.36)^i^	2.9	Exp 5	<.001
11	BiLSTM or LSTM^j^	Structured+Llama 3 summaries+temporal	0.38 (0.37-0.38)^k^	2.6	Exp 9	<.001

a Column values are formatted as text representation or temporal representation.

b SSI: surgical site infection.

c Not applicable.

d CIs are not reported for the manual review estimates, as these values are derived from published average processing times aggregated across multiple institutions.

e RF: random forest.

f FFNN: feed-forward neural network.

g UMLS: Unified Medical Language System.

h BiLSTM: bidirectional long short-term memory networks.

i Precision at 0.9 recall values with statistical significance (*P*<.05).

j LSTM: short-term memory networks.

k Highest precision at 0.9 recall values.

Overall, we found that clinical utility tracked overall performance well. Our best deep learning model (Table 3: Exp 11) achieved higher clinical utility than our best RF models (Table 3: Exp 3). The addition of temporal information led to a slight increase in clinical utility (Table 3: Exp 11 vs Exp 9). Neural models achieved higher clinical utility than published gold-standard conventional ML models (Table 3: Exp 5 vs Exp 3). However, harnessing the ability of LLMs to understand complex contextual underpinnings is required to achieve substantial performance gains over conventional approaches (Table 3: Exp 11 vs Exp 3). This would entail a 1.5-fold improvement (2.6 charts reviewed per SSI detected with our method vs 4 charts reviewed per SSI detected with published methods [12,13]) in the efficiency of surveillance over published gold-standard automated surveillance systems. It would also enable approximately a 13-fold increase (2.6 charts reviewed per SSI detected with our method vs 33 charts reviewed per SSI detected with manual chart review [13]) in the number of SSIs that can be detected and the effective number of charts that can be reviewed in the same time over manual surveillance alone.

## Discussion

### Principal Findings

In this study, we developed and evaluated an automated SSI surveillance system using multimodal EHR data from approximately 30,000 surgical procedures across 2 health care facilities. Our approach integrated structured clinical data, temporal information, and clinical text using deep learning models, while leveraging generalist foundation models to summarize clinical documentation. The best-performing architecture combined foundation model–based text summarization with recurrent neural networks (BiLSTM and LSTM) that captured contextual patterns in clinical notes and temporal dependencies in structured clinical data. This approach achieved a precision of 0.38 at a recall of 0.9. Additionally, inclusion of data from multiple hospitals and surveillance programs may improve the generalizability of the model by exposing it to broader clinical variability.

These findings suggest that multimodal, data-driven deep learning approaches can improve the performance of automated SSI surveillance systems compared with previously reported rule-based or conventional ML methods. Two key implications follow from this observation. First, improvements in performance appear to scale with the availability of large clinical datasets. Over the past 2 decades, health care systems have generated rapidly increasing volumes of digital clinical data, including structured EHR data and clinical documentation [21]. Data-driven approaches that leverage these large datasets may therefore enable increasingly effective surveillance systems capable of monitoring a larger proportion of surgical procedures.

Second, deep learning approaches reduce the need for labor-intensive rule design and manual feature engineering, which have historically been required for rule-based or traditional ML surveillance systems [22]. By learning patterns directly from multimodal data, these models can simplify system development and potentially reduce the cost and effort required to maintain surveillance tools as clinical workflows evolve.

Recent advances in LLMs and generalist foundation models also create new opportunities for extracting meaningful information from clinical text. Previous research has shown that the use of LLMs can improve the efficiency of SSI surveillance methods for some procedure types [23-25]. These models contain broad linguistic and domain knowledge that allow them to perform many NLP tasks with minimal task-specific training [26]. Our results suggest that using foundation models selectively for tasks where they demonstrate strong capabilities—such as summarizing clinical documentation—can enhance downstream prediction tasks when combined with deep learning architectures designed to model temporal clinical data.

### Comparison With Prior Work

Automated SSI surveillance systems are most useful when deployed as a screening step prior to manual chart review, allowing infection prevention personnel to focus on cases most likely to involve infection. In this setting, systems must maintain high recall to ensure that true SSI cases are not missed while maximizing precision to minimize the number of charts requiring manual review.

Our best-performing model achieved a precision of 0.38 at a recall of 0.9, representing approximately a 1.5-fold improvement in precision compared with previously reported automated surveillance approaches, which achieved precision levels of 0.24 to 0.28 at similar recall thresholds. In practical terms, this improvement would substantially reduce the number of charts that infection prevention staff must review to identify each SSI case.

When applied at scale, improvements in precision of this magnitude could significantly expand the number of surgical cases that can be monitored through surveillance programs while simultaneously reducing the manual review workload. Estimates based on national surgical volumes suggest that such improvements could translate into substantial reductions in annual manual review hours for infection prevention personnel (see Multimedia Appendix 4 for detailed calculations).

### Limitations

Our study has the following limitations. First, although our dataset comprises data from 2 facilities, both are affiliated with the same health system, University of Washington Medicine. However, the procedure types included at each hospital differ substantially, and therefore the 2 sites are not directly comparable for the purposes of stratified performance reporting. As a result, we did not report model performance stratified by hospital. This distinction also limits our ability to assess site-level variability in model performance within this study.

More broadly, this also constrains the generalizability of our findings. Because the data were derived from a single health system, the model may reflect shared characteristics such as documentation practices, clinical workflows, and EHR infrastructure. Accordingly, the performance of our model in external settings—particularly in institutions with different EHR vendors, surveillance protocols, or documentation patterns—remains uncertain and will require validation in independent cohorts. Future studies should evaluate the transportability of this approach across diverse health care systems and EHR environments.

We did not leverage prompt engineering approaches with LLMs, which would enable completely zero-shot approaches requiring no training samples. In our approaches, we used BiLSTM and LSTM models as part of our architecture, which require training. Future work could explore the performance of such approaches on SSI prediction. In addition, technologies like retrieval-augmented generation might improve intrainstitutional performance. Finally, we excluded imaging data as a modality, though it could enhance performance by confirming SSI-negative cases or identifying non-SSI infections. Future work will focus on standardizing image formats and extracting relevant features to improve predictions [27]. Recently, vision-aligned LLMs may also hold promise in this regard and will be explored as well. Lastly, it is important to mention that while automated surveillance methods can mitigate certain aspects of bias in the manual review process, they can potentially also introduce new bias caused by biases in the training data used to train such models and other factors.

### Conclusions

Automated surveillance approaches using ML can enable more efficient SSI surveillance. This can enable more robust analyses of surveillance data and more informed quality improvement initiatives targeting SSI rates.

## Supplementary material

10.2196/87896 Multimedia Appendix 1 Expanded data representation.

10.2196/87896 Multimedia Appendix 2 Examples of clinical text excerpts which drove model predictions towards a positive or negative classification for models using UMLS concept sentences.

10.2196/87896 Multimedia Appendix 3 Examples of clinical text excerpts which drove model predictions towards a positive or negative classification for models using summarized clinical notes.

10.2196/87896 Multimedia Appendix 4 Calculation details on how surveillance efficiency improvement with our study was calculated.

## Abbreviations

BiLSTM: bidirectional long short-term memory network
CNN: convolutional neural network
EHR: electronic health record
HIPAA: Health Insurance Portability and Accountability Act
HMC: Harborview Medical Center
LLM: large language model
LSTM: long short-term memory network
ML: machine learning
NHSN: National Healthcare Safety Network
NLP: natural language processing
NSQIP: National Surgical Quality Improvement Program
RF: random forest
SSI: surgical site infection
TF-IDF: term frequency-inverse document frequency
UMLS: Unified Medical Language System
UWMC: University of Washington Medical Center
WBC: white blood cell
