# Supplementary material for: Automated Identification of Surgical Site Infections From Electronic Medical Records: Retrospective Observational Predictive Modeling Study
Source: JMIR Perioper Med. 2026 Jun 26;9:e87896. doi: 10.2196/87896 (PMC13311363; doi:10.2196/87896)
Supplement: Multimedia Appendix 1 [file periop-v9-e87896-s001.docx]

## Expanded Data Representation

Structured data representation:

Structured data elements included (1) surgical procedure characteristics, (2) laboratory values (e.g., WBC counts and microbiology orders), (3) postoperative fever measured by temperature, (4) antibiotic administration events, and (5) postoperative consultations with infectious disease teams or interventional radiology for drain placement. Surgical procedure type was one-hot encoded as “Surgical Procedure Class.” Each case was labeled based on whether a reoperation occurred within 90 days (“Reoperation”).

Consultation variables were represented as binary indicators for postoperative infectious disease consultation (“ID Consult”) or interventional radiology drain placement (“IR Drain”) within 90 days after surgery. Additional binary indicators captured whether a culture order for wound, tissue, fluid, or cerebrospinal fluid (“Culture Ordered”) or administration of SSI-relevant antibiotics (“Antibiotic Administration”) occurred within the same postoperative window.

Continuous laboratory values and vitals, including WBC counts and body temperature, were summarized using their maximum values within the first 90 postoperative days (White Blood Cell_max postop and Temperature_max postop). Outliers (e.g., >100k/mm³ for WBC or >50°C for temperature) and missing values were replaced with the mean from the training set. All values were then min–max normalized based on the training set before model input.

Unstructured clinical note representation: The clinical text corpus included all clinical notes associated with a surgical case from 7 days before surgery to 90 days afterward, totaling 3,193,094 notes. The five most frequent note types were Nursing Record/Note (Inpatient) (499,963), Telephone Encounter (179,769), Progress Note (115,118), Operative Report (47,555), and Discharge Summary (34,465). Patient portal messages were not included in unstructured notes. For each surgical case, all notes were concatenated into a single pseudo-document representing the case’s text data.

Pseudo-documents were preprocessed as follows: (1) text was lowercased; (2) stopwords, defined by the NLTK stopword list[18], were removed for approaches using discrete text vectorization (approaches 1 and 2 below); and (3) numbers and punctuation were removed for those same approaches.

A key challenge was that clinical notes contained substantial information unrelated to surgical site infection (SSI), making the SSI signal relatively sparse. At the same time, relevant information could appear across diverse note types and at different points in the postoperative period. To address this, some text representations were designed to extract information most relevant to SSI prior to model input (denoted as “information extracted” below).

Text representation and vectorization approaches:

1. Unigrams: Each word was treated as a feature using a bag-of-words representation with term frequency–inverse document frequency (TF-IDF) weighting[19]. Only words appearing in at least five documents were included. The top N features were selected using an ANOVA F-test prior to model training, with N determined through hyperparameter search.
2. Unified Medical Language System (UMLS) disease and chemical concepts: Disease and chemical concepts were defined using UMLS semantic groups[20] and identified with scispaCy[21]. We retained the 200 concepts with the highest ANOVA F-values relative to the SSI label. These concepts were represented using a bag-of-words TF-IDF approach, where document frequency was defined by the number of surgical cases containing the concept. Remaining TF-IDF implementation details matched the unigram approach.
3. Sentences containing UMLS disease and chemical concepts (information extracted): To capture contextual information, we extracted sentences containing identified disease or chemical UMLS concepts. Sentences were ordered temporally by note date and by their position within each note. To reduce noise, only sentences containing concepts appearing in fewer than 15% of pseudo-documents were retained. The resulting sentence sequences were vectorized using word embeddings trained on our dataset with the word2vec Continuous Bag of Words architecture[22]. In a separate approach, we also used embeddings derived from ClinicalBERT, which incorporates domain-specific knowledge through pretraining and fine-tuning on clinical corpora[23].
4. Clinical note summaries (information extracted): We utilized Llama-3-70B-Instruct (Meta AI), accessed through the Hugging Face Transformers framework, to summarize clinical notes. The average length of each clinical note in our dataset was 1,772 words. We condensed each note into a summary of 100 words. The manuscript authors, two of which are clinicians, jointly reviewed generated summaries to ensure they were consistent, reproducible, accurate, and without hallucinations. Using parallel GPU inference (4 parallel GPU’s), the average processing time was approximately one second per note, and the full summarization process for our 3,193,094 notes was completed in about nine days. These summaries for each clinical note were concatenated temporally in order of note date. Our summarization prompt was as follows: *“Summarize the following clinical note emphasizing development of infection and administration of antibiotics for surgical site infection. Limit the summary to less than 100 words.”* Our constrained prompt (“emphasizing development of infection and administration of antibiotics”) was designed to limit hallucination. To capture information more salient to SSI prediction, we only summarized notes containing disease or chemical UMLS concepts. We vectorized the sequence of clinical note summaries using word embeddings.

Temporal Data Representation: To assess the effect of adding temporal data on automated SSI identification performance, we enriched our structured data representation of laboratory values (WBC count, absolute neutrophil count, and vitals (temperature)) by using the time stamps associated with each measurement or test. In approaches incorporating temporal information, we excluded these laboratory values and vitals from our static structured features (excluded White Blood Cellmax postop and Temperaturemax postop defined in Structured data representation Section). We extracted temporal data from the 7-day preoperative to 90-day postoperative period. The preoperative look-back period was designed to capture signals related to preexisting infections. We aggregated our data by computing the daily maximum for each variable (**Figure 1**). To standardize the temporal representation for each patient, we fixed the sequence length by padding shorter sequences with zeros until they matched the maximum length (ten timepoints). This length of ten was determined through hyperparameter tuning. We chose zero padding because SSI-negative cases in our dataset had lower laboratory values, fewer vital measurements, and higher rates of missing data compared to positive cases.

*Figure 1: Method for constructing our temporal representation.* *TP = time point.

For each laboratory value or vital, we constructed 4 features to capture the following important signals:

1. The fold change above the upper limit of the normal range that the measured value has on a particular day: We defined fold change as the value recorded for the test or vital sign divided by the upper limit of the normal range for that test or vital (upper limit for WBC count: 10.5*10^3^/mm^3^, upper limit for ANC:7*10^3^/mm^3^, upper limit for temperature: 38^o^C based on literature review). High values of laboratory tests or vitals may be indicative of infection.
2. The number of days between consecutive recordings of a laboratory value or vital: A laboratory test ordered on consecutive days may indicate an ongoing clinical suspicion of infection.
3. The number of days between the date of surgery and the measurement date: Different indications for the ordering of laboratory tests (e.g., urinary tract infection, SSI) occur at different timepoints before or after surgery.
4. Whether the value is higher than the normal range: Our reasoning behind including this variable was similar to that for measurement representation #1.

To construct these features, we used four different transformations of the temporally ordered sequence of each laboratory value or vital (**Figure 1**). We vertically stacked the sequences for all of the four features derived from WBC count, ANC, and temperature to constitute the temporal input to our CNN or LSTM models.
