# Supplementary figures and images for: Automated Identification of Surgical Site Infections From Electronic Medical Records: Retrospective Observational Predictive Modeling Study
Source: JMIR Perioper Med. 2026 Jun 26;9:e87896. doi: 10.2196/87896 (PMC13311363; doi:10.2196/87896)

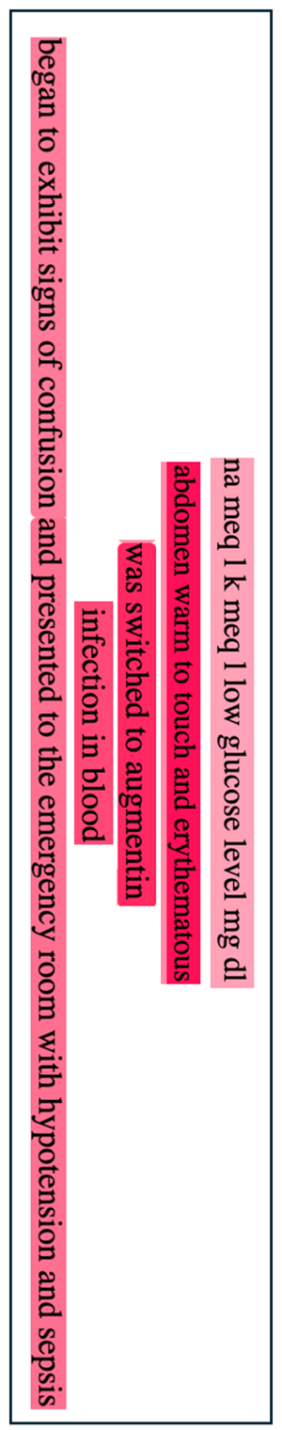

Supplement: Multimedia Appendix 2 [file periop-v9-e87896-s002.png]

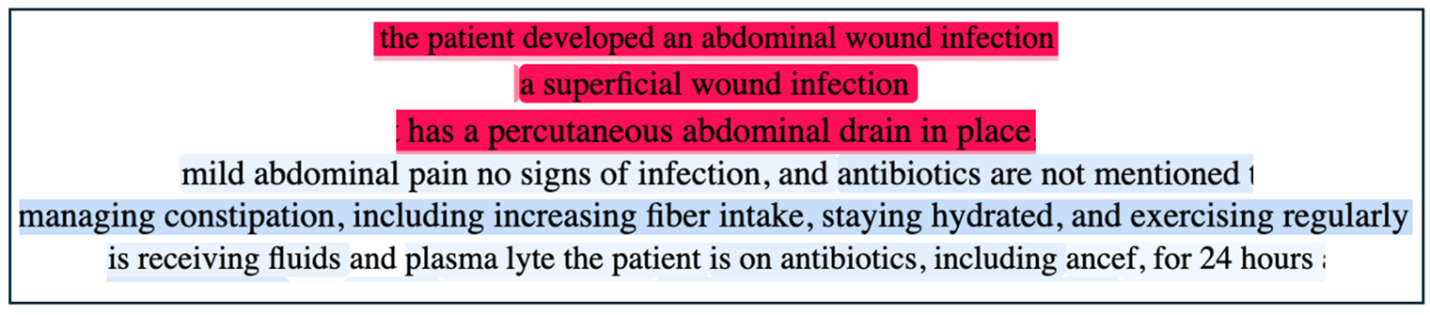

Supplement: Multimedia Appendix 3 [file periop-v9-e87896-s003.png]

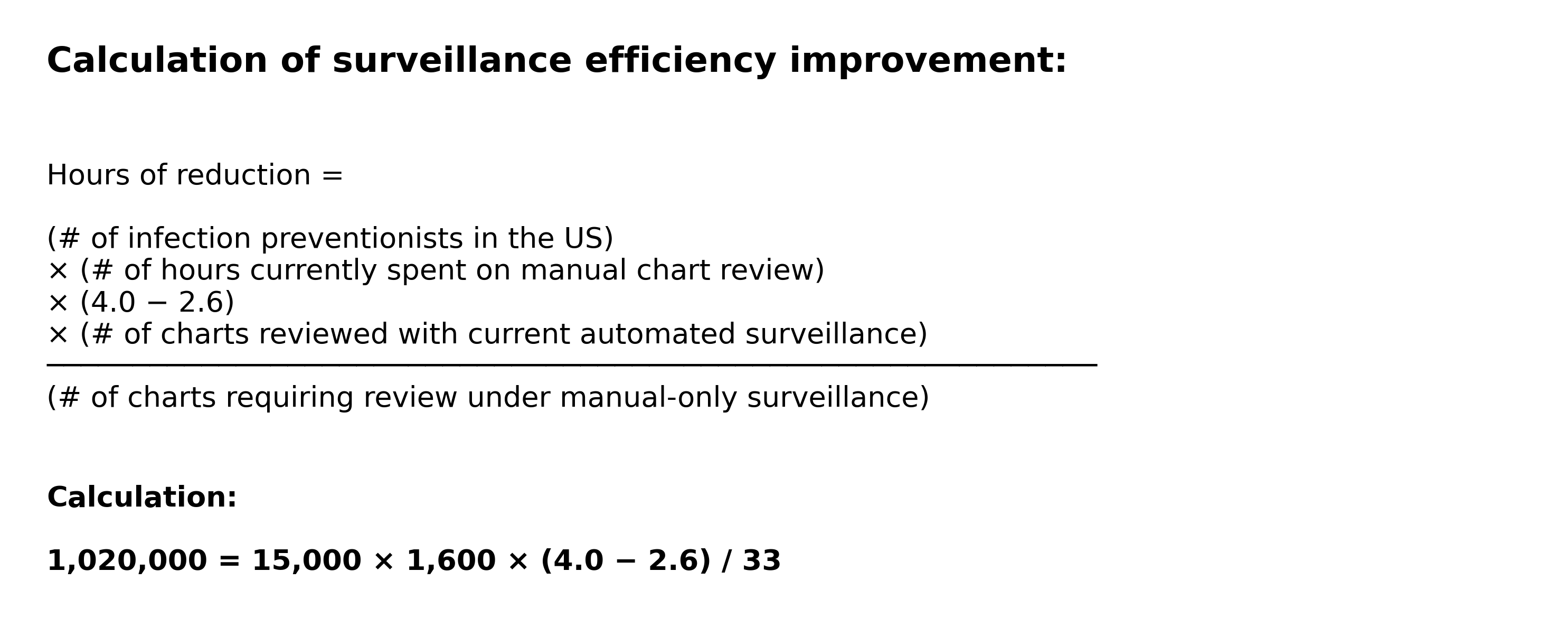

Supplement: Multimedia Appendix 4 [file periop-v9-e87896-s004.png]
